# Effect of surface pretreatments on surface roughness and repair bond strength of aged machinable hybrid ceramic with nanohybrid resin composite

**DOI:** 10.1038/s41598-025-20403-0

**Published:** 2025-09-24

**Authors:** Sara Abdelhady, Hamdi Hamama, Jukka P. Matinlinna, Salah Mahmoud

**Affiliations:** 1 Conservative Dentistry Department, Faculty of Dentistry, Mansoura University, Mansoura, Egypt; 2 Faculty of Oral and Dental Medicine, Alsalam University, Tanta, Egypt; 3 Biomaterials Science, Division of Dentistry, The University of Manchester, Manchester, M13 9PL UK

**Keywords:** Repair, CAD/CAM hybrid ceramic, Adhesion strength, Surface pretreatment, Microshear bond strength, Engineering, Materials science

## Abstract

To evaluate the repair bond strength of a nanohybrid resin composite to aged machinable hybrid ceramic after using three surface pretreatments. Specimens of the hybrid ceramic blocks were grouped into four groups based on the ceramic surface pretreatment: no treatment control group, bur grinding group, HF etched group, and grit-blasting group. All the groups were tested for surface roughness by an optical profilometer. Test groups were subdivided into immediate and aged groups based on aging protocol. A nanohybrid resin composite was placed onto prepared resin/ceramic hybrid material surfaces for the micro shear bond strength test. Deboned specimens were inspected using a stereomicroscope and a scanning electron microscope to determine the failure mode. Data was analyzed using ANOVA and the Tukey’s HSD test. One-way ANOVA test for surface roughness showed no significant difference among the differently pretreated surfaces. Two-way ANOVA after aging indicated significant differences of surface pretreatments and aging protocol on MSBSof the nanohybrid composite bonded to hybrid ceramic (*p* < 0.05). Surface pretreatments increased the bond strength of resin composite bonded to hybrid ceramic material. Aging had a detrimental effect on MSBSof nanohybrid resin composite to hybrid ceramic.

## Introduction

Advancements in software, hardware, and materials have led to the development of computer-aided design/computer-assisted machining (CAD/CAM) technologies. These technologies now provide technicians and dentists with multiple alternatives for designing dental restorations^1^. Developing new durable and aesthetic restorative materials to meet patients criteria for aesthetics and biocompatibility had become the demand of all restorative clinicians^2^. Modern ceramics are based on the concept of composite resins^3,4^. These ceramics can be classified into: resin-based ceramics (RBCs), polymer-infiltrated ceramic networks (PICNs), or hybrid ceramics depending on their composition and manufacturing process^5,6^. Moreover, they are newly developed materials with polymer-like qualities such as minimal antagonist wear and improved flexural properties, as well as color stability^7^.

These materials offer several shades and translucency options, they also are highly polishable, have low abrasiveness, and are easy to mill and repair^8^. In addition, they provide a significant benefit over glaze firing due to their resin matrix composition, allowing for more rapid completion of the restoration and its easier polishing to achieve smooth and lustrous natural looking surfaces^9^.

On the other hand, ceramic restorations in clinical practice are prone to fractures because traditional dental ceramics are inherently brittle^5,10^. Other reasons for fractures of even failure, include interceramic imperfections, trauma, or parafunctional habits^11^. Understandably, replacing a failed restoration may not be the most cost-effective alternative and must not lead to excessive loss of tooth structure^12^. Intraoral repair is an effective alternative to restorations’ replacement for localized defects that are no longer clinically acceptable or tolerable^13^. This process is safe, cost-effective, and time-saving, it even reduces the danger of traumatic damage to teeth and promotes the longevity of dental restorations^14^.

Intraoral repair is a form of minimally-invasive procedure that involves adding a new restorative material to the existing failed restoration, either with or without prior preparation^15^. However, repairing a failed ceramic restorations is a complex clinical issue as clinicians struggle to choose an accurate ceramic repair system that produces reliable findings^16^. Nevertheless, there is still limited documentations on the clinical performance, longevity and success of repaired restorations^17^. Whereas, for standard cementation in clinical practice there are widely accepted guidelines for diverse ceramic materials in prosthodontics^18,19^.

Researches have devised and assessed various repair techniques to improve the performance, durability, and aesthetics of ceramic restorations with a demand of expanding the surface area to improve wettability and reduce surface tension^20^. Repair incorporates both chemical and mechanical approaches like acid etching technique that can be performed using hydrofluoric acid, acidulated phosphate fluoride, or phosphoric acid^21–23^. Sandblasting with aluminum oxide or silica-coating^24–27^. In addition, also bur grinding is available: it is a low-cost, simple, and quick technique that does not require chemical agents or other equipment. However, there is currently no consensus on the most effective repair strategy for achieving satisfactory clinical results as it depends on multiple factors such as the kind of ceramic, repair process, aging condition, and resin composite type so the ideal repair protocol to treat cracked or chipped restoration is still debatable^28,29^.

The current laboratory study aimed to evaluate the mechanical and chemical surface pretreatments on surface roughness and repair bond strength of aged CAD/CAM hybrid ceramic with nanohybrid resin composite. Consequently, this study was conducted to test the null-hypothesis of that there was no significant difference in surface roughness of the CAD/CAM hybrid ceramic either treated mechanically or chemically. Also, this study was performed to test the null-hypothesis of that there was no significant difference in microshear bond strength (MSBS) of resin composite to the CAD/CAM hybrid ceramic after mechanical or chemical surface pre-treatments. Furthermore, the last null-hypothesis was that aging had no significant effect on MSBS of resin composite to CAD/CAM hybrid ceramic.

## Materials and methods

The current laboratory study analyzed the MSBS(adhesion strength) of three repair protocols on a CAD/CAM hybrid ceramic. The compositions, brands, and manufacturers of all materials used in this laboratory study are all presented in Table 1.

**Table 1:** Materials used in the study.

Material	Porcelain Etchant™	Porcelain Primer™	Shofu™ HC Hard	Single Bond™ Universal	Tetric™ *N*-Ceram resin composite
Specification	Hydrofluoric acid-etch	Pre-hydrolyzed silane primer	Resin Nanoceramic	Universal adhesive	Nanohybrid resin composite
Manufacturer	Bisco, Schaumburg, Illinois, USA	Bisco, Schaumburg, Illinois, USA	Shofu Dental, Ratingen, Germany	Tetric N-Bond Ivoclar Vivadent, Schaan, Liechtenstein	IvoclarVivadent, Schaan, Liechtenstein
Batch number	2,400,000,483	2,300,111,902	0819919	Z03WDZ	Z03KBY
Chemical Composition	9.5% hydrofluoric acid gel.	Silane with methacrylate (1–10%), acetone (30–70%) and ethanol (30–70%).	Silica powder, micro fumed silica, zirconium silicate fillers 61% by weight. UDMA, TEGDMA.	Methacrylates, ethanol, water, highly dispersed silicon dioxide, initiators and stabilizers.	Dimethacrylates (19–20 wt%). The fillers contain barium glass, ytterbium trifluoride, mixed oxide and copolymers (80–81 wt%).

### Methods

#### Specimen Preparation and grouping

The statistical software G*Power was utilized for estimating the appropriate sample size^30^ Based on a level of significance of 0.05, an effect size of 0.5, and a power of 80, it was determined that a sample size of 18 specimens per group were required.

Eight blocks (10 mm x 12 mm x 16 mm) of Shofu™ hybrid ceramic were randomly divided into four study groups based on the type of surface pretreatment provided to each block surface. Next, each group was further divided into two sub-groups based on the artificial aging protocol: immediate group and aged group after aging. The pretreatments were caried out by one operator to the ceramic surfaces before composite resin application.

- Control group: The specimens remained intact, serving as the study’s control.
- Bur grinding group: a green-banded diamond fissure bur was used with a high-speed rotary tool under water cooling for 4 s to roughen the surface manually by the operator.
- Hydrofluoric acid group: 9.5% hydrofluoric acid, HF, was applied for 90 s, followed by 60 s of water spray. Following that, the surface was cleansed with an ultrasonic cleaner for five minutes before air drying and with a fine microbrush. The silane coupling agent (one drop) was then applied evenly across the surface and left in place to react for 60 s.

Al_2_O_3_ grit-blasting group: The specimens were gently grit-blasted for 20 s with 50 μm Al_2_O_3_(Ney, Blastmate II™, Yucaipa, Canada) under 2 bar pressure using slowly rotating motion and a constant perpendicular distance of 1 cm. Specimens were placed in a specific holder that formed right angle and had a 10 mm gap between the nozzle and the surface. The specimens were next rinsed with distilled water and allowed to air-dry for 60 s^31^.

#### Surface roughness

Following surface pretreatments, surface roughness was assessed for each specimen by an optical profilometer machine (U500x Digital Microscope, Guangdong, China). On the specimens, a quantitative surface treatment analysis was carried out using a 3D-surface analyzer equipment. A USB digital microscope with a camera was used to take pictures of the specimens. For each surface treatment, a mean value was obtained resembling the average of three measurements to define the roughness profile. The average Ra values corresponded to the mean of peaks and valleys.

### MSBS

#### Application of repair Nano-hybrid resin composite

A heavy body silicone-based impression material was used to construct a cylinder with a 2 mm thickness at the edges, a 1 mm thickness at the ceramic rectangular section, and a diameter of 2.5 cm. Each ceramic rectangle was accommodated by a depression on the inside surface of the rubber base cylinder, which would assist in the creation of resin composite micro cylinders. The marks were created in the depressions of the rubber foundation cylinders and placed evenly according to the diameter of each rectangle. Then, these marks were drilled with a 1.0 mm diameter cylindrical bur, each resulting hole was 1.0 mm in diameter, and the gap between each hole and the subsequent hole was 2.0 mm, creating a mold that would securely hold the composite micro cylinders. A universal adhesive resin (Tetric N-Bond IvoclarVivadent) was applied to the blocks surfaces then Light cured for 20–30 s. The already made rubber base cylinders were realigned over their blocks, and the resin composite material (Tetric™ N-Ceram, Ivoclar Vivadent, Schaan, Liechtenstein) was condensed and Light-cured for 20–30 s into the cylindrical holes. Each ceramic block surface had nine composite micro cylinders (*n* = 9). The exact identical procedure was utilized to prepare all of the specimens.

#### Artificial aging protocol

**Fig. 1:**
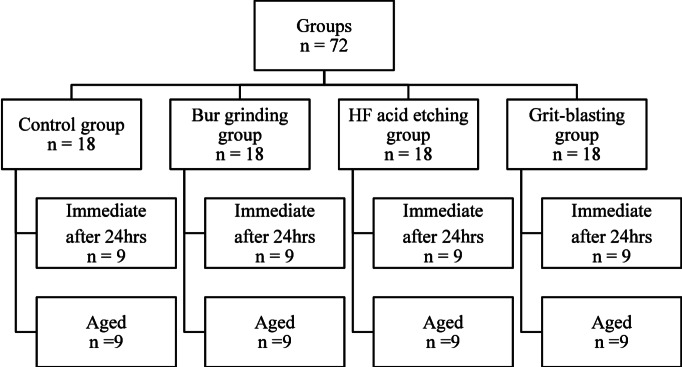
Schematic diagram showing the study design and study groups.

The ceramic/composite blocks were randomly categorized into two study groups: one half of the specimens were assessed immediately after 24 h, and the other half were subjected to an artificial aging protocol to simulate a long-term service (Fig. 1).

Artificial saliva storage: the specimens were aged in artificial saliva at 37 °C for 6 months in an incubator to mimic the heat fluctuations that occur in the oral environment. The artificial saliva was (Artificial Saliva Gal Fovet; SAGF) medium (NaCl 125.6 mg L^−1^; KCl 963.9 mg L^−1^; KSCN 189.2 mg L^−1^; KH_2_PO4 654.5 mg L^−1^; urea 200.0 mg L^−1^; NaSO_4_.10H2O 763.2 mg L^−1^; NH_4_Cl 178.0 mg L^−1^; CaCl_2_.2H2O 227.8 mg L^−1^; NaHCO_3_ 630.8 mg L^−1^).

Thermo-cycling: each specimen was placed in an automatic thermal cycling machine and cycled for 2000 cycles between thermostatically controlled water baths of 5 °C (± 1 °C) and 55 °C (± 1 °C) with a dwell period 30 s. The transfer time between the two bathtubs was 15 s^32^.

#### Microshear bond strength

**Fig. 2:**
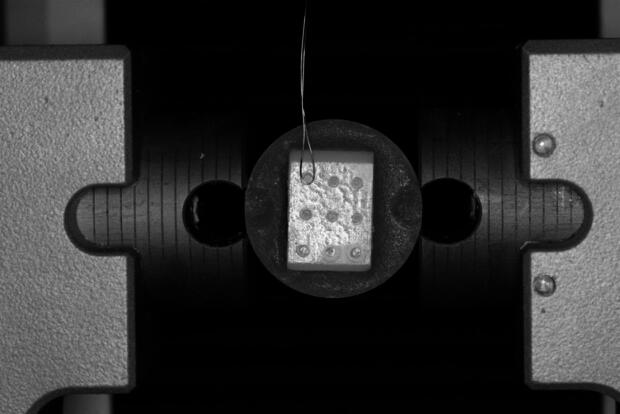
Photograph showing the universal testing machine and specimens.

Each block was next positioned on the machine’s lower jig. A stainless-steel wire was wrapped around the resin composite micro-cylinder at the ceramic-composite interphase and connected to the upper moving arm of the universal testing machine. Each cylinder specimen received a shear force of 0.5 mm/min cross-head speed until failure occurred. The MSBS was calculated by dividing the maximum load at failure (N) by the bonding area (mm2) and measured in MPa using computer software (NexygenMT Llyod Instruments) (Fig. 2).

#### Failure mode analysis

To determine the failure mode, all specimens were inspected with a stereomicroscope (Olympus model SZ2-ILST, Tokyo, Japan) at x12 magnification immediately after fracture. The failure modes were classified as follows: (a) adhesive failure (resin composite restoration and CAD/CAM hybrid ceramic blocks interface), and (b) mixed failure (adhesive and cohesive failure). Fractured blocks representing the types of failure in each group were cleansed with ethanol and air-dried for 1 min. Then, they were mounted on metal stubs, gold sputtered (SPI Module - Sputter Carbon/Gold Coater, EDEN instruments, Japan) and inspected with Scanning electron microscope (SEM) (JSM6510LV, JEOL, Tokyo, Japan) at x1,500 magnification.

## Results

### Surface roughness

**Fig. 3:**
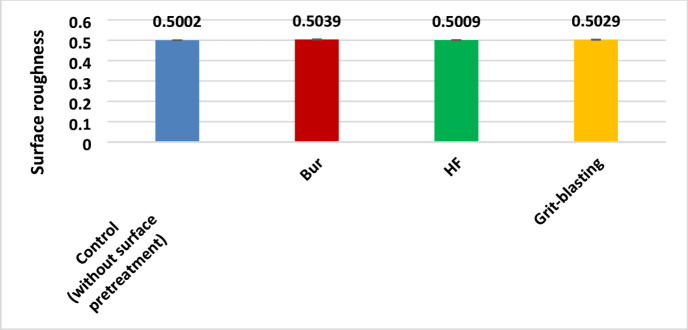
Results of One-way ANOVA test of surface roughness means among test groups.

In terms of surface pretreatment approach, RA values were calculated as the average of each treated surface’s peaks and valleys. The control group had the lowest mean surface roughness value, whereas the bur group had the highest one as shown in Fig. 3. There is no significant difference in the surface roughness between the different surface pretreatments test groups.

### MSBS

**Fig. 4:**
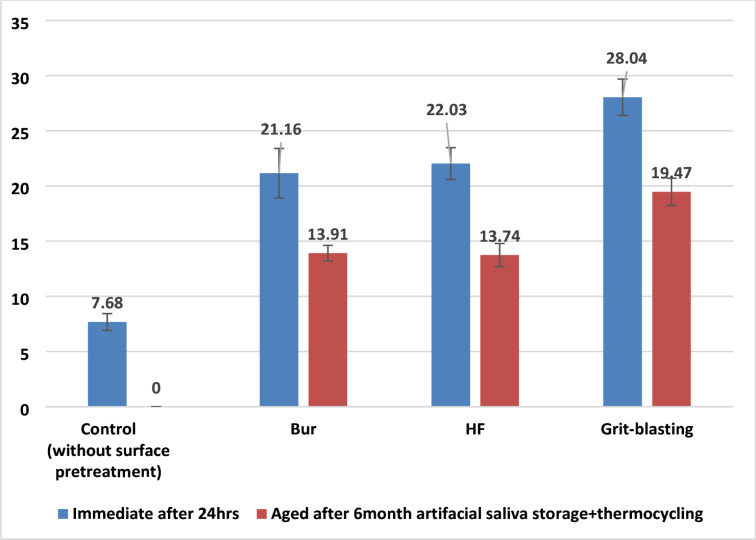
Mean and standard deviation values of MSBS test among test groups and multiple comparisons between means.

The Kolmogorov-Smirnov and Shapiro-Wilk tests revealed that all MSBS data had a normal distribution pattern (*p* < 0.05). As a result, a parametric two-way ANOVA test was conducted. The two-way ANOVA test showed that surface pretreatment and time have a significant effect on MSBS (*p* < 0.05). The MSBS mean values and standard deviations for all groups, as well as the Tukey’s HSD *post hoc* multiple comparisons between groups, are shown in Fig. 4.

The Tukey’s HSD *post hoc* multiple comparison test revealed that surface pretreatments significantly raised the MSBS of materials compared to control groups without surface pretreatments (p < 0.05). The control group, ‘Immediate’ was showing 7.68 ± 2.28 MPa, and ‘Aged’ 0.00 ± 0.00 MPa, had significantly lower mean values compared to all other test groups (p < 0.05). The MSBS mean value of the bur group: ‘Immediate’ 21.16 ± 6.74 MPa and ‘Aged’ 13.91 ± 2.14 MPa exhibited a significant difference in contrast to the other test groups (p < 0.05). The MSBS mean value of ’HF group’, ‘Immediate’ 22.03 ± 4.32 MPa and ‘Aged’ 13.74 ± 3.16 MPa differed significantly from the other test groups. The MSBS mean values of the grit-blasting groups: ‘Immediate’ 28.04 ± 4.93 MPa and ‘Aged’ 19.47 ± 3.71 MPa revealed a significant difference in comparison to the other test groups (*p* < 0.05) with the highest mean values.

### Pearson correlation test between surface roughness and microshear bond strength

Pearson correlation coefficient was used to assess correlation between continuous parametric data as shown in Table 2.

A statistically significant positive correlation (*R* = 0.712) was found between surface roughness and MSBS for the HF group. And for the bur and grit-blasting groups there was negative correlation found between MSBS and surface roughness.

**Table 2:** Pearson correlation test between surface roughness and microshear bond strength.

			MSBS	Surface roughness
Control	MSBS	Pearson Correlation	1	0.071
	Surface roughness	Pearson Correlation	0.071	1
Bur	MSBS	Pearson Correlation	1	− 0.562
	Surface roughness	Pearson Correlation	− 0.562	1
HF	MSBS	Pearson Correlation	1	0.712^*^
	Surface roughness	Pearson Correlation	0.712^*^	1
Grit-blasting	MSBS	Pearson Correlation	1	− 0.184
	Surface roughness	Pearson Correlation	− 0.184	1

### Failure mode analysis

**Fig. 5:**
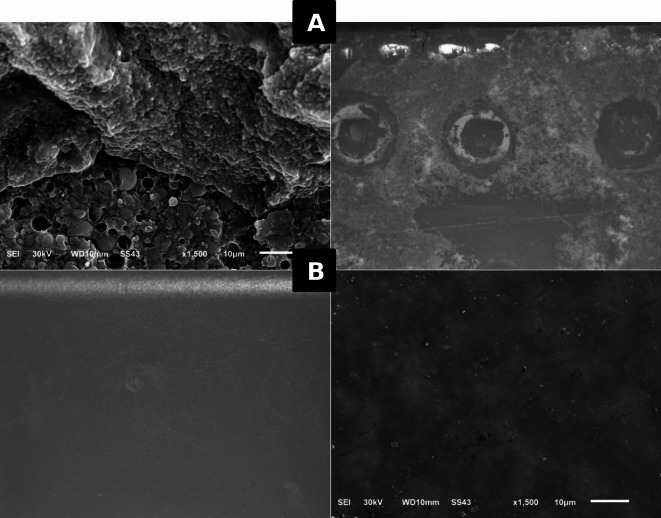
Micrographs showed mode of failure by a stereomicroscope (x12) and a scanning electron microscope (x1,500): **(A)** mixed failure, and **(B)** adhesive failure.

**Fig. 6:**
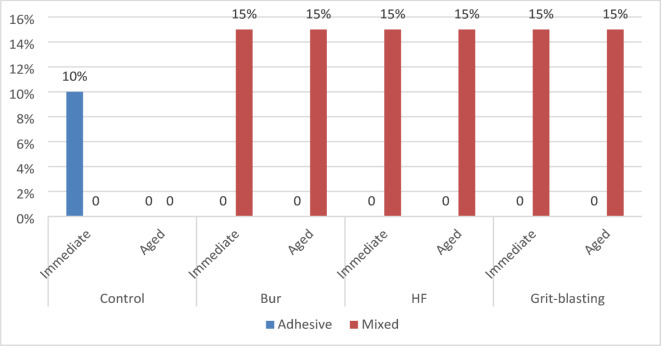
Failure mode distribution and percentages of each type of failure at each group of specimens in comparison to the other tested study groups from the total amount of specimens.

The findings of the failure mode analysis for all specimen groups with diverse surface pretreatment protocols revealed that all the test groups showed mixed failure except for the control groups, had shown adhesive failure type. The various types of failure modes are shown in (Figs. 5 and 6) using a stereomicroscope and scanning electron microscope respectively.

## Discussion

It is well and widely acknowledged that intraoral repair is a minimally invasive and cost-effective technique. This current study was conducted to evaluate the intraoral reparability of one CAD/CAM hybrid ceramic material that comprises the clinical benefits of ceramics and resin composites^12^. The ceramic material employed in this study was Shofu™ HC hybrid ceramic. Shofu™ block HC is an innovative hybrid material includes a resins matrix with dispersed fillers, containing 61% zirconium silicate integrated in a polymer matrix created at high-temperature and high-pressure. It possesses the advantages of the ease and rapidity of milling and polishing, and it is amicable to the opposing dentition^33^.

The repair strength of restorative materials is mostly determined by mechanical interlocking, exactly as in dentin bonding^34^. Various surface pretreatment methods had been applied to ceramic material surfaces, grit-blasting (with 50 μm Al_2_O_3_) creates a wide surface area with increased topography. This is highly important for wettability and a micro-retentive structure for micromechanical locking of luting cement materials, thereby enhancing adhesion strength^35^. The primer was ‘only’ added to HF-treated in order to avoid the deleterious effect on MSBS, which is attributed high acidic environment resulted from reaction between acidic MDP-containing adhesive and HF treated surface^36,37^. studies. Furthermore, the primer was not used with the other surface treatment methods to avoid masking effect for combined values which might influence the outcome of this laboratory studies.

Grinding with a diamond bur as a mechanical treatment to increase the bonding of repair resin composite and ceramics is available, but it is not without risks. It may be described as a simple and inexpensive way for mechanical treatment of the ceramic surface, but requires a well-trained operator with an observant eye^38^. As for the sake of standardization and crack prevention the bur grinding was carried out in one stroke at one direction for 4 s. These methods were, at least in principle, designed to prevent surface cracks and deficiencies while ensuring a homogeneous surface treatment^39^. However, with traditional ceramics that wouldn’t apply as they are inherently brittle materials and mechanical roughening methods would have limitations. For ceramics and some polymers that are susceptible to acid, etching with HF (e.g., buffered hydrofluoric acid) produces a roughened surface^10,32^. However, for the used shofu HC hard blocks a higher concentration and longer time of etching is recommended to enhance the roughness as it is composed of resin matrix with dispersed ceramic fillers that make the surface more resistant to hydrofluoric acid etching. On the contrast, the baseline for this study is the control group, which did not receive any surface pretreatment. It should be clarified that ‘untreated’ control groups were selected to obtain basic comparable information without interfering the bonding substrate to clearly understand the effect of study variables (Surface treatment protocol)^40–42^. These methods are frequently used for surface treatments during intraoral ceramic restoration repair^43,44^. The universal adhesive system used in this study is said to have a unique chemistry that comprises MDP, i.e., 10-methacryloyloxydecyl dihydrogen phosphate, in addition to some other components. Given that, it allows the adhesive to chemically react and attach to glass ceramic surfaces without the need for a separate ceramic primer^45^. Hybrid ceramics consist of a resin matrix embedded with ceramic fillers containing metal oxides, MDP’s have the ability to chemically bind to the metal oxides within the composition of hybrid ceramics^36,37^.

The current laboratory study focused on the adhesion strength of roughened surfaces, which plays a pivotal role for the bonding of restorative materials. Diamond-burred surfaces exhibited the highest roughness values, followed by grit-blasted surfaces. Surfaces that had not been pretreated and those that had been etched with HF had the lowest roughness levels. Although, the recorded surface roughness test results in this current investigation showed disparities across the tested groups, there was no statistically significant difference between them. Therefore, the null hypothesis, that there was no significant difference in surface roughness among variously treated hybrid ceramic surfaces, was accepted. Accordingly, surface roughness alone should not be used to justify differences in bond strength values.

The molds for resin composite micro-cylinders were created using a silicone-based impression material that does not adhere or stick to the resin composite. This enables easy removal of the molds while maintaining the structural stability of the micro-tubes. Tetric™ N Ceram resin composite is a nanohybrid direct resin composite made up of some dimethacrylates (19–20 wt%). The fillers include barium glass, ytterbium trifluoride, mixed oxide, and some copolymers (80–81 wt%). This resin composite was chosen because of its specific filler technology, which offers low shrinkage stress and high aesthetic effects, making it suitable for anterior and posterior direct restorations as well as intraoral repairs of failed crowns and bridges. It was also chosen in this study because of its potential for chipped restorations intraoral repair and high bond strength, which is consistent with Kalra et al^46^.

To evaluate the adherence of resin composites, MSBS test was opted and performed in the study since the specimens could be easily prepared and help to avoid the cohesive fracture of numerous samples^47^. The MSBS test is commonly thought to be straightforward and easy to perform. However, the reliability of this method is called into question because of the uneven stress distribution in the bond area^48^. Moreover, all shear bond strength tests suffer from the plain fact that they, ironically, cannot measure shear forces at all. One improvement suggested and already applied in dental research is the so-called enclosed-mold microshear bond strength, also studied by Zakir et al.^49,50^. In the current investigation, MSBS data revealed that the untreated control group exhibited the lowest bond strength due to the lower surface roughness in contrast to pretreated ceramic surfaces, resulting in less mechanical interlocking and good wettability of adhesive systems. Grit-blasting has often demonstrated statistically significant higher MSBS values. This is understood to be attributed to increased surface roughness, which increases interlocking between resin composite and substrate as well as increased surface energy that enabled for optimal chemical bonds. Bur grinding and HF etching yielded much lower bond strength than grit-blasting surface pretreatment.

The MSBS outcome of current study was in agreement with results of Şişmanoğlu et al.^30^although they compared various hybrid ceramic materials. On the other hand, Güngör et al.^51^. reported that grit-blasting resulted in a lower bond strength of resin composite to hybrid ceramic material than pretreating with diamond bur grinding. This could be attributed to changes in bond strength testing methodologies, grit-blasting pressure, and time compared to this study. Nevertheless, Silva et al.^52^. revealed that bur grinding yielded higher bond strength values than grit-blasting this could be attributed to the use of different diamond bur grain sizes (180 μm) and Al_2_O_3_ particle sizes (45 μm) may have an impact on bond strength tests. In this in vitro investigation, surface treatments considerably increased the MSBS of resin composite to CAD/CAM hybrid ceramic materials, which is consistent with earlier results^29,34,51^.

Dental restorations are subjected to various temperatures in the mouth cavity. The temperature of the oral cavity normally fluctuates between 35 and 37 °C, but this can vary based on diet and beverages^53^. Temperature fluctuations can eventually cause mechanical stresses, cracking, and spreading in resin-containing materials because of the variances of the thermal expansion in fillers and the matrix^54,55^. As a result of natural aging in a humid and thermally active oral environment, restorations frequently fail. Aging of the restoration should be considered when planning restoration repairs^56^. Thermal cycling is an accelerated artificial aging strategy for dental materials that has been done to imitate intraoral temperature fluctuations, in which thermal strain on the bonding surface is induced by the influence of liquids and thermal change^57^. In the current investigation, the control group’s specimens failed during thermo-cycling, demonstrating the stress and alterations caused by thermal cycling on the recovered surfaces of restorative materials. Analysis of pretest failures revealed that all failures occurred in the untreated control group.

Furthermore, as shown in the MSB Sresults above, in the immediate and aged samples of the same surface treatment group, there was a decline in the adhesion strength values in the aged groups after the artificial aging protocol, which supports the fact that aging had a detrimental effect on microshear bond strength. The failure mode analysis of debonded specimens confirmed the microshear test findings. Control groups with the lowest bond strength values demonstrated more adhesive failures, while grit-blasting groups with the highest bond strength values had the most mixed failures. The mixed failure pattern indicated higher adhesion, however the adhesive failure pattern typically corresponds to poor binding strength values^58,59^.

In light of the outcome of this study, the tested null-hypothesis that there was no significant difference in MSBS of resin composite to the CAD/CAM hybrid ceramic after mechanical or chemical surface pre-treatments was rejected. Furthermore, the null hypothesis that aging had no significant effect on MSBS of resin composite to CAD/CAM hybrid ceramic was also rejected.

The current study encountered some limitations; The use of single type of materials for both hybrid ceramic and resin composite. As the type of resin composite impacts its bond strength to hybrid ceramics. Additionally, inclusion of failed specimens prior to the MSBS test is another limitation of the current study as failed specimens should be eliminated from statistical analysis, this should be taken into account while evaluating the outcomes.

## Conclusions

This laboratory investigation found that several surface treatment approaches seemed to improve the microshear adhesion strength between resin composite and a hybrid ceramic. Grit-blasting was the most effective method for improving the bond strength between resin composite and hybrid ceramic.

## Data Availability

The datasets used and/or analysed during the current study are available from the corresponding author on reasonable request.
